# Advancements in Immunology and Microbiology Research: A Comprehensive Exploration of Key Areas

**DOI:** 10.3390/microorganisms12081672

**Published:** 2024-08-14

**Authors:** Angel Justiz-Vaillant, Darren Gopaul, Sachin Soodeen, Chandrashekhar Unakal, Reinand Thompson, Shalini Pooransingh, Rodolfo Arozarena-Fundora, Odalis Asin-Milan, Patrick Eberechi Akpaka

**Affiliations:** 1 Department of Para-Clinical Sciences, University of the West Indies, St. Augustine Campus, St. Augustine 00000, Trinidad and Tobago; sachin.soodeen@my.uwi.edu (S.S.); chandrashekhar.unakal@sta.uwi.edu (C.U.); reinand.thompson@sta.uwi.edu (R.T.); shalini.pooransingh@sta.uwi.edu (S.P.); patrick.akpaka@sta.uwi.edu (P.E.A.); 2 Port of Spain General Hospital, University of the West Indies, St. Augustine Campus, St. Augustine 00000, Trinidad and Tobago; darren.gopaul2@my.uwi.edu; 3 Eric Williams Medical Sciences Complex, North Central Regional Health Authority, Champs Fleurs 00000, Trinidad and Tobago; rodolfo.fundora@sta.uwi.edu; 4 Department of Clinical and Surgical Sciences, Faculty of Medical Sciences, University of the West Indies, St. Augustine 00000, Trinidad and Tobago; 5 Independent Researcher, Laval, QC H7E 2Z8, Canada; odalis_asin@yahoo.es

**Keywords:** bacterial proteins, extended-spectrum beta-lactamases (ESBLs), immunology, microbiology, NPSLE, experimental vaccines, tuberculosis, global healthcare

## Abstract

Immunology and microbiology research has witnessed remarkable growth and innovation globally, playing a pivotal role in advancing our understanding of immune mechanisms, disease pathogenesis, and therapeutic interventions. This manuscript presents a comprehensive exploration of the key areas in immunology research, spanning from the utilisation of bacterial proteins as antibody reagents to the intricate realms of clinical immunology and disease management. The utilisation of bacterial immunoglobulin-binding proteins (IBPs), including protein A (SpA), protein G (SpG), and protein L (SpL), has revolutionised serological diagnostics, showing promise in early disease detection and precision medicine. Microbiological studies have shed light on antimicrobial resistance patterns, particularly the emergence of extended-spectrum beta-lactamases (ESBLs), guiding antimicrobial stewardship programmes and informing therapeutic strategies. Clinical immunology research has elucidated the molecular pathways underlying immune-mediated disorders, resulting in tailored management strategies for conditions such as severe combined immunodeficiency (SCID), neuropsychiatric systemic lupus erythematosus (NPSLE), etc. Additionally, significant efforts in vaccine development against tuberculosis and HIV are highlighted, underscoring the ongoing global pursuit of effective preventive measures against these infectious diseases. In summary, immunology and microbiology research have provided significant contributions to global healthcare, fostering collaboration, innovation, and improved patient outcomes.

## 1. Introduction

Immunology research has experienced significant growth worldwide, catalysing breakthroughs in understanding immune mechanisms, disease pathogenesis, and therapeutic interventions [1]. This comprehensive literature review navigates through a diverse array of immunological investigations, spanning from innovative applications of bacterial proteins as antibody reagents [2] to the intricate realms of clinical immunology and disease management [3], with a particular emphasis on pivotal research domains, such as engineering chimeric proteins for immunodiagnosis [4], vaccine development [5], and clinical studies targeting diseases like African swine fever virus [6] and HIV [7].

Additionally, the emergence of extended-spectrum beta-lactamases (ESBLs) poses a significant threat to healthcare systems worldwide, necessitating concerted efforts to understand and combat antimicrobial resistance [8]. This introduction outlines the current research endeavours aimed at addressing critical priorities in global healthcare, including antimicrobial resistance [9] and the development of advanced immunological techniques [10].

Significant advancements in immunological techniques and blood banking practises have not only enhanced diagnostic and therapeutic capacities [11] but have also improved patient care standards and safety protocols globally [12]. Furthermore, this introduction explores the intricate journey of navigating clinical immunology, encompassing a spectrum of immune-mediated disorders and complex diseases, such as chronic granulomatous disease [13], transient hypogammaglobulinemia of infancy [14], neuropsychiatric systemic lupus erythematosus [15], severe combined immunodeficiency [16], developments in HIV vaccines and challenges [17], and messenger RNA (mRNA) vaccines [18]. Additionally, investigations concerning antibiotic resistance [19] and tuberculosis vaccine [20] shed light on the comprehensive nature of immunological and microbiological research globally.

This introduction sets the stage for a comprehensive exploration of the diverse facets of immunology and microbiology, reflecting on the pivotal role of these disciplines in advancing scientific knowledge and clinical care paradigms on a global scale. The subsequent sections delve deeper into specific research areas, highlighting their implications for global healthcare and emphasising the collaborative efforts aimed at improving patient outcomes worldwide. As the field of immunology continues to evolve, fuelled by ongoing research and innovation, its impact on global health remains profound. By fostering collaboration and innovation, immunology research continues to play a pivotal role in addressing healthcare challenges and improving the lives of individuals worldwide.

## 2. Unveiling the Potential of Bacterial Proteins as Antibody Reagents and Engineering Chimeric Proteins

**Figure 1:**
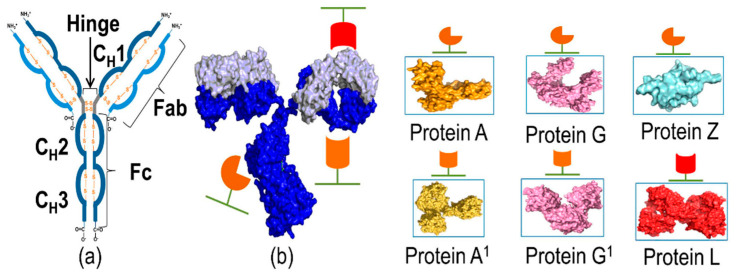
(**a**,**b**) An illustration of an antibody and the most common bacterial proteins which recognise the antibody. The figure was taken from [21].

Bacterial immunoglobulin-binding proteins (IBPs) are a diverse group of molecules known for their unique ability to interact with immunoglobulins. Among the well-studied IBPs are protein A (SpA) from *Staphylococcus aureus* [21], protein G (SpG) from *Streptococci* [22], and protein L (SpL) from *Peptostreptococcus magnus* [23]. The design of chimeric IBPs allows for customisation for specific applications, enhancing their performance in diagnostic tests and research experiments. Chimeric IBPs can improve the accuracy and efficiency of immunodetection assays and facilitate the purification of immunoglobulins for various uses [24]. Figure 1 shows the IPBs which recognise the human IgGs [21].

One remarkable feature of IBPs is their broad specificity in binding to immunoglobulins [25]. They can interact with a wide range of mammalian and non-mammalian immunoglobulins, including IgG [24]. This interaction does not interfere with the antigen-binding sites, making IBPs powerful tools in immunological assays and diagnostic tests. Proteins like SpA and SpG have been extensively used in serological assays to diagnose infectious diseases in both humans and animals, such as assays to detect *Borrelia burgdorferi*, the causative agent of Lyme disease [26,27].

IBPs hold immense potential beyond diagnostics, including biomedical research, therapy, and biotechnology. Their ability to selectively bind to immunoglobulins has paved the way for novel immunodiagnostic techniques, protein engineering, and purification processes. Studies have investigated the reactivity of IBPs (SpA, SpG, and SpL) and recombinant protein LA with immunoglobulins from various avian and mammalian species. The findings show that SpLA exhibited the highest reactivity, while SpL was the least reactive. IBPs can aid in diagnosing infectious diseases and purifying immunoglobulins [24].

These proteins are intricately woven into the cell walls of microorganisms, serving as potent tools for evading host immune responses while offering remarkable binding affinity to a wide spectrum of immunoglobulins [28,29]. Innovations in this field have addressed issues such as host–cell proteins (HCPs) co-eluting with IgG during protein A chromatography, enhancing IgG purity by minimising HCP contamination and protein A leaching during the elution process [30].

SpA binds primarily to the Fc region of IgG, specifically at the CH2-CH3 junction, and can also bind to the Fab regions of some VH3-type immunoglobulins [31,32]. IBPs enhance the diagnostic specificity of antibodies by selectively binding to particular immunoglobulin types or subclasses, allowing for more precise detection and characterisation of antibodies. This specificity reduces background noise and cross-reactivity, improving the accuracy of assays [33].

Marine mammals have experienced a rise in infectious diseases, such as brucellosis, morbillivirus, herpesvirus, and poxvirus. Serological diagnostic methods, like ELISA, immunofluorescence assays, and Western blotting, are used to detect antibodies against these pathogens. However, the limited availability of commercial secondary antibodies for marine mammals has led researchers to explore proteins A and G and chimeric protein AG as alternatives. Studies show that these proteins effectively detect marine mammal immunoglobulins, enhancing the development of serological assays for diagnosing infectious diseases in marine mammals [34].

The advent of chimeric proteins heralds a new era in immunodiagnosis, characterised by enhanced specificity, stability, and diagnostic accuracy. Through the fusion of protein domains from disparate sources, researchers have developed next-generation immunodiagnostic tools. Chimeric IBPs and other molecules meticulously engineered to optimise binding kinetics and broaden target specificity hold immense promise in early disease detection and precision medicine [35,36,37].

## 3. Illuminating Pathways in Vaccine Development and Clinical Studies

### 3.1. An HIV Experimental Vaccine

Research on the idiotypic network in Leghorn laying hens revealed that inoculating hens with a 35.5 kD outer membrane protein of *Pasteurella multocida* (Pm35.5), its idiotype (Id), or Pm35.5 anti-Id led to the presence of specific antibodies in egg yolks. These antibodies demonstrated the significant inhibition of Pm35.5 binding, indicating potential for anti-idiotypic vaccine development [38].

A study immunised brown Leghorn layer hens with HIV-1 viral peptides to elicit strong anti-HIV immune responses. Feeding chicks with hyperimmune eggs from these hens induced the production of anti-anti-idiotypic antibodies capable of neutralising HIV, suggesting a novel approach for HIV immunotherapy. Further studies are required to validate these findings in humans [39].

Previous research identified immunogenic peptide motifs within HIV envelope glycoproteins gp120 and gp41, showing promise as vaccine candidates. However, developing an HIV vaccine remains challenging due to the virus’s rapid mutation and diversity, which complicates the elicitation of broadly neutralising antibodies (bnAbs) [40].

### 3.2. African Swine Fever Virus Vaccine Update

African swine fever virus (ASFV) poses a significant threat to the global swine industry, with mortality rates up to 100% in domestic pigs. Despite extensive research, effective ASF vaccines remain elusive. Inactivated ASFV vaccines fail to induce protective immunity, and subunit vaccines have proven ineffective. Only live attenuated vaccines (LAVs) have shown high efficacy, with one approved in Vietnam. However, these LAVs demonstrate a delayed onset and short duration of protection. ASFV’s unique characteristics, such as immune evasion and multiple distinct infectious virions, significantly impede vaccine development [41,42,43].

### 3.3. Vaccine Development Faces Challenges in Inducing Strong Immune Responses

Despite the success of vaccines and therapeutic antibodies, developing new drug candidates is laborious, time-consuming, costly, and risky. Vaccine development faces challenges in inducing strong immune responses across diverse populations and protecting against highly variable pathogens. Advances in high-throughput sequencing and structural biology have provided insights into germline immunoglobulin genes and antibodies, revealing their associations with antigens and disease manifestations. These insights are crucial for improving antibody screening and developability [44,45].

### 3.4. New Developments in HIV Vaccines and Challenges

**Figure 2:**
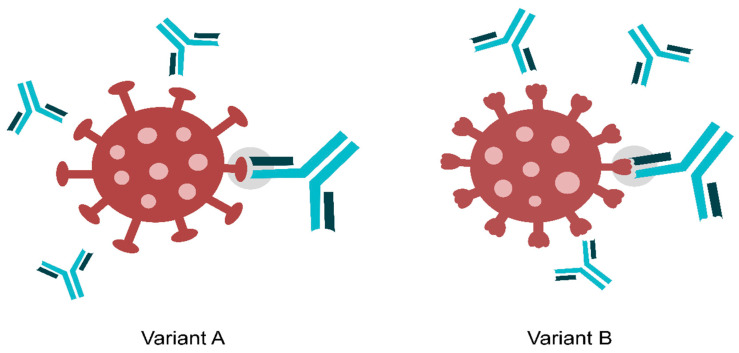
The neutralisation of two viral variants. The humoral immune response is specific and reacts against multiple epitopes. The licence was purchased from shutterstock.com.

Developing an HIV vaccine involves designing immunogens to elicit bnAbs. Stabilised, cleaved Env trimers and sequential boosting with Env variants are considered promising strategies. Germline antibody reactivity to immunogen templates is crucial, and modifications may enhance binding. Recent advances in single-cell antibody cloning uncovered new bnAbs with increased potency and breadth. Clinical trials with bnAbs like 3BNC117 have shown promise in reducing viral loads, and passive infusion studies in macaques demonstrated protection against SHIV challenges. Immunisations with multi-clade Env-derived trimers aim to drive antibody maturation towards neutralisation breadth, incorporating stabilising mutations to enhance immunogenicity [46,47,48]. Figure 2 shows a picture of an IgG-mediated viral neutralisation. 

### 3.5. Tuberculosis Vaccines

Tuberculosis (TB) remains a global health concern. The Bacillus Calmette–Guérin (BCG) vaccine is the only licenced TB vaccine, providing effective protection to infants and children against severe forms of the disease. However, its efficacy in adults is inconsistent. New TB vaccine candidates, including whole-cell vaccines, adjuvanted protein subunit vaccines, and viral vector-delivered subunit vaccines, are undergoing clinical trials. These candidates aim to prevent TB in adolescents and adults, serve as BCG boosters, or reduce TB therapy duration. Recent studies suggest that central memory T cells and locally secreted IgA might correlate with protection, highlighting the need for identifying such correlates in future clinical trials [49,50,51].

### 3.6. Messenger RNA (mRNA) Vaccines

The success of mRNA vaccines against COVID-19 has prompted pharmaceutical and biotech companies to explore their application across various diseases. They offer several advantages over traditional vaccines, such as utilising body cells to induce both innate and adaptive immunity and enabling rapid, large-scale production due to efficient in vitro transcription. Although mRNA vaccines face challenges such as poor stability and strong immunogenicity, advancements in modifications and delivery methods have mitigated these issues. Consequently, mRNA vaccines are a promising option for preventing and treating various diseases [52,53].

mRNA vaccines have also shown potential in treating cancers and autoimmune diseases. For instance, DC-based mRNA vaccines targeting melanoma demonstrated early success in reducing lung metastases in a mouse model. BioNTech AG has developed personalised mRNA vaccines targeting breast cancer tumour antigens and neoantigens, showing promising immune responses in preliminary trials. Additionally, modified mRNA vaccines have been effective in mouse models of autoimmune diseases like multiple sclerosis, reducing disease progression and promoting regulatory T cells. mRNA vaccines also offer a safer approach to preventing allergies by encoding allergens and inducing Th1 cell responses, generating long-term memory responses without booster vaccinations [52,54].

## 4. Microbiological Insights and Antimicrobial Resistance Surveillance

### 4.1. Extended-Spectrum Beta-Lactamases (ESBLs): A Global Public Health Challenge

Extended-spectrum beta-lactamases (ESBLs), enzymes produced by bacteria within the Enterobacteriaceae family, confer resistance to a range of beta-lactam antibiotics. These enzymes, encoded by mobile genetic elements, facilitate transfer between bacterial strains and species, complicating treatment strategies due to their ability to hydrolyse various beta-lactam antibiotics [55,56]. ESBLs are classified by molecular structures (Ambler classification) or functional systems (Bush–Jacoby–Medeiros) into types like SHV, TEM, and CTX-M, each with distinct biochemical characteristics and resistance profiles. Accurate detection methods, like PCR and sequencing, are crucial for identifying ESBL-producing organisms, aiding in surveillance and infection control [57,58]. Global epidemiological studies highlight ESBLs’ widespread presence across clinical and environmental settings, underscoring the need for enhanced surveillance and intervention strategies [57,59]. 

### 4.2. Types of ESBL and Mechanisms of Resistance

ESBLs confer resistance by hydrolysing the beta-lactam ring, essential for the bactericidal activity of these antibiotics. Variants like SHV, TEM, and CTX-M each have unique hydrolytic capabilities. SHV-1 beta-lactamases hydrolyse narrow-spectrum cephalosporins and penicillin, while SHV-2 and SHV-3 extend this activity to broad-spectrum cephalosporins. CTX-M enzymes target a wide range of cephalosporins and aztreonam. OXA-type beta-lactamases, though typically weak against ESBL activity, include variants like OXA-48 that resist carbapenems. These enzymes are often plasmid-encoded, facilitating rapid spread among bacterial populations [60,61,62,63,64,65,66].

### 4.3. Detection of ESBLs in Medical Institutions

Jamaican research identified the clonal persistence of ESBL-producing *K. pneumoniae* in hospitals, suggesting endemic presence without significant outbreaks [67]. In Northwest Mexico, ESBL-producing *E. coli* predominantly carried the blaCTX-M-15 gene, with widespread dissemination among clinical and community settings [68]. Additional studies in Brazil and Morocco have underscored the global spread and high resistance rates of ESBL-producing bacteria, necessitating ongoing monitoring and intervention efforts [69,70,71]. 

### 4.4. Methicillin-Resistant Staphylococcus aureus (MRSA) in the Caribbean and Globally

Limited data on MRSA in the Caribbean indicate diverse MRSA and MSSA lineages. In Trinidad and Tobago, MSSA predominates, with concerns over PVL-positive community-acquired strains, while MRSA exhibits diverse strains, including ST239-MRSA-III. In Jamaica, MRSA prevalence is relatively low, with commonly identified strains such as ST8-MRSA-IV, USA300, and ST5/ST225-MRSA-II. A DNA microarray-based analysis classified clinical *S. aureus* isolates from Trinidad and Tobago and Jamaica, filling a critical gap in understanding the epidemiology of *S. aureus*/MRSA in the Caribbean [72]. In Brazil, the distinction between healthcare-associated MRSA (HA-MRSA) and community-associated MRSA (CA-MRSA) is blurring, with CA-MRSA being increasingly found in hospitals. Traditionally, HA-MRSA is multidrug-resistant, while CA-MRSA is often susceptible to non-β-lactam antibiotics; however, this resistance pattern is changing [73]. Surveillance is essential to understand MRSA’s dissemination and resistance patterns. A bivalent vaccine developed in a murine model shows promise against *S. aureus*, inducing strong protective immunity with a combination of neutralising and opsonic antibodies and memory T cells [74].

### 4.5. Mechanisms of Bacterial Resistance

Bacterial resistance mechanisms include beta-lactamase production, alterations in penicillin-binding proteins (PBPs), and efflux pump overexpression. Combination therapy with beta-lactam antibiotics and beta-lactamase inhibitors is crucial but faces challenges with emerging enzyme variants. Quinolone resistance arises from mutations in DNA gyrase and topoisomerase IV, reducing the drug binding efficacy. The overexpression of efflux pumps expels quinolones and aminoglycosides, lowering intracellular drug concentrations. Gram-negative bacteria employ efflux pumps like AcrAB-TolC and form biofilms, which impede antibiotic diffusion and increase resistance. Advancing single-molecule techniques could enhance the understanding of biofilm resilience and antimicrobial resistance. A study on *Salmonella enterica* isolates from raw chicken revealed discrepancies between whole-genome sequencing (WGS) predictions and actual antimicrobial resistance (AMR) phenotypes, highlighting heteroresistance and WGS limitations in detecting resistance mechanisms. Additionally, tetracycline-resistant lactic acid bacteria strains in fermented products demonstrated resistance mediated by ribosomal protection proteins on mobile genetic elements. These mechanisms enable bacteria to evade antibiotics, posing significant challenges in treating infections and necessitating ongoing research on novel therapeutic strategies [75,76,77,78,79,80,81].

## 5. Evolution of Immunological Techniques and Advancements in Blood Banking

According to the World Health Organization (WHO), nearly 120 million units of blood are donated globally each year. Despite this large number, there remains a shortfall in meeting the global demand for safe and timely blood transfusions. Blood donation rates vary significantly across different regions, with high-income countries collecting up to seven times more donations per population than low-income countries. Ensuring the safety and quality of donated blood is crucial, and challenges include inadequate screening for infectious diseases such as HIV, hepatitis B, and hepatitis C, especially in low-income countries [11,82].

The authors of this study conducted a follow-up survey in Trinidad and Tobago to delve into the dynamics of the knowledge, attitudes, and practises surrounding blood donation. With a sample size ranging from 349 to 356 participants, this study aimed to track changes over time in the community’s perception and behaviour towards blood donation. This study emphasises the importance of ongoing education and blood safety and encouraging regular blood donation [83].

Ensuring the safety of blood product transfusions is paramount in modern healthcare, considering the potential risks associated with transfusion-related complications. Safety measures encompass multiple stages, from donor screening to recipient monitoring, all aimed at minimising adverse events and maximising the benefits of transfusion therapy. Donor screening protocols are rigorously implemented to reduce the risk of transmitting infectious agents such as HIV, hepatitis B and C, and syphilis. Sophisticated testing methods, including nucleic acid testing, have significantly enhanced the detection of viral markers, further bolstering the safety of blood products. Additionally, blood establishments adhere to strict regulations and guidelines established by regulatory bodies like the Food and Drug Administration and that formerly known as the American Association of Blood Banks to uphold the highest standards of donor selection and testing. During processing and storage, blood products are meticulously handled to maintain their integrity and minimise the risk of bacterial contamination or degradation. Advanced technologies, such as leukoreduction, which involves filtering out white blood cells, have been shown to mitigate transfusion-related complications, such as febrile non-haemolytic reactions and alloimmunisation. Furthermore, recipient monitoring plays a crucial role in ensuring transfusion safety. Healthcare providers assess patients for signs of transfusion reactions and closely monitor vital signs during and after transfusion, and previous transfusion history is also considered to tailor transfusion strategies and minimise risks. In summary, the safety of blood product transfusion is upheld through stringent donor screening, meticulous processing and storage procedures, adherence to regulatory standards, and vigilant recipient monitoring. These measures collectively mitigate the risk of transfusion-related complications and contribute to the overall efficacy and safety of transfusion therapy [84].

## 6. Immunological Techniques’ Impact on Global Health and the Support of Quantitative Data

Some authors claim that immunological techniques have had a revolutionary impact on global health, but they fail to provide supporting quantitative data. They argue that advances in immunology have significantly improved the prevention, diagnosis, and treatment of diseases worldwide. They highlight the development of vaccines, which have drastically reduced the incidence of infectious diseases, such as polio, measles, and influenza. Furthermore, they mention that immunological research has led to a better understanding and management of autoimmune diseases and allergies, improving the quality of life for millions. These authors also point out that immunotherapy has become a promising approach to cancer treatment, offering new hope to patients. Despite these assertions, the lack of empirical evidence and specific examples to substantiate their claims weakens their argument. More concrete data and case studies would strengthen their position and provide a clearer picture of the actual impact of immunological techniques on global health [85,86].

Immunological research has also made significant strides in combatting autoimmune diseases. The introduction of biological therapies has transformed the treatment landscape for conditions like rheumatoid arthritis and multiple sclerosis, markedly improving patient outcomes. A study published in *The Lancet* indicated that biologics can reduce disease activity and improve physical function in 60–70% of patients with rheumatoid arthritis who do not respond to traditional therapies [87].

In oncology, immunotherapy has emerged as a ground-breaking treatment. The development of immune checkpoint inhibitors, such as nivolumab and pembrolizumab, has shown remarkable efficacy in treating various cancers, including melanoma and non-small-cell lung cancer. Clinical trials have demonstrated that these therapies can achieve long-term remission in a subset of patients, with one study reporting a five-year survival rate of 34% for patients with advanced melanoma treated with nivolumab [88].

Furthermore, advancements in immunodiagnostic techniques have enhanced disease detection and monitoring. For example, enzyme-linked immunosorbent assays (ELISAs) are widely used to detect HIV, allowing for early diagnosis and timely intervention. The global scale-up of HIV testing has been pivotal, with UNAIDS reporting that 84% of people living with HIV knew their status by the end of 2019. These examples underscore the profound impact of immunological techniques on global health, highlighting the need for ongoing research and investment in this critical field [89].

Advancements in immunodiagnostic techniques have significantly enhanced disease detection and monitoring, leading to improved health outcomes. Immunodiagnostic tests, such as enzyme-linked immunosorbent assays (ELISAs), have become pivotal in the early diagnosis of diseases like COVID-19, confirmed by polymerase chain reaction (PCR), enabling timely intervention and treatment. Additionally, immunodiagnostic techniques have revolutionised cancer diagnostics. Biomarkers, such as prostate-specific antigen (PSA) for prostate cancer and cancer antigen 125 (CA-125) for ovarian cancer, are used in immunoassays to monitor disease progression and response to therapy. These techniques provide non-invasive, rapid, and accurate results, significantly aiding in early detection and personalised treatment plans. Furthermore, advancements in point-of-care testing (POCT) using immunodiagnostic methods have improved disease management in remote and resource-limited settings. POCT devices enable immediate testing and results, facilitating prompt medical decisions and reducing the burden on healthcare facilities [85,86,88].

## 7. Navigating Clinical Immunology: From Bench to Bedside Management

### 7.1. Severe Combined Immunodeficiency Disorders

Severe combined immunodeficiency (SCID) is a group of rare, life-threatening disorders characterised by a profound impairment of the immune system. SCID is classified into different types based on the genetic mutations underlying the condition, with each one presenting unique challenges in diagnosis and management. The microbiological associations of SCID, including susceptibility to various pathogens due to compromised immunity, are discussed, shedding light on the infectious risks faced by individuals with this condition. Additionally, this paper explores treatment strategies for SCID, which typically involve haematopoietic stem cell transplantation (HSCT) or gene therapy to restore immune function. This publication provides valuable insights into the classification, microbiological aspects, and therapeutic options for SCID, contributing to the understanding and management of this severe immunodeficiency disorder [90,91,92].

In addition to the provided information, SCID is characterised by a primary inherited immunodeficiency that typically manifests before three months of age and can lead to fatal outcomes. The condition arises from a deficiency in both T and B cell function, resulting in susceptibility to opportunistic infections caused by various pathogens, such as bacteria, viruses, fungi, and protozoa. SCID presents in autosomal, X-linked, and sporadic forms, with early signs including recurrent infections and lymphopenia. Prompt immunological investigation is crucial upon the suspicion of SCID, allowing for timely diagnosis and intervention. Stem cell transplantation stands as the primary treatment modality, aiming to restore immune function. This review aims to provide a comprehensive understanding of the microorganisms associated with SCID and their management. It delineates SCID as a syndrome and outlines the diverse range of microorganisms affecting affected children, along with approaches for investigation and treatment [90].

### 7.2. Transient Hypogammaglobulinemia of Infancy

Transient hypogammaglobulinemia of infancy (THI) is a primary immunodeficiency characterised by a temporary decline in serum IgG levels, typically occurring in infants aged 5 to 24 months. Preterm infants are especially vulnerable due to the insufficient transfer of IgG across the placenta. This systematic review aimed to assess the diagnostic criteria for THI. A total of 16 studies out of 215 identified articles were eligible, with bias assessed across six domains. A total of 31% of the studies had a low bias risk, 25% had a high risk, and 44% were unclear. THI diagnosis is confirmed only after IgG levels normalise, indicating that it is not benign and necessitating monitoring for recurrent infections. The diagnostic criteria should consider vaccine and isohaemagglutinin responses to distinguish THI from other infant immunological disorders [93,94].

### 7.3. Chronic Granulomatous Disease (CGD)

Chronic granulomatous disease (CGD) is a primary immunodeficiency resulting from mutations in genes encoding the subunits of the NADPH oxidase enzyme complex, impairing the phagocytic function of the innate immune system. This review aims to comprehensively address the pathogens associated with CGD and its management. Patients, typically children, with CGD face recurrent life-threatening infections and potential infectious or inflammatory complications. Management strategies involve antibacterial prophylaxis with trimethoprim–sulfamethoxazole; antifungal prophylaxis, typically with itraconazole; and interferon gamma immunotherapy to reduce infection risk. Haematopoietic stem cell transplantation (HCT) is the preferred treatment for CGD, offering successful outcomes [95,96,97,98].

### 7.4. Neuropsychiatric Systemic Lupus Erythematosus (NPSLE)

Systemic lupus erythematosus (SLE) is a chronic autoimmune disease affecting various organs, including the nervous system. Its aetiology involves environmental, genetic, and immunological factors, leading to autoantibody production against self-antigens. Failure in self-tolerance mechanisms in T and B cells contributes to tissue damage. Diagnosis remains challenging despite available criteria. Neuropsychiatric manifestations, termed neuropsychiatric SLE (NPSLE), lack definitive pathological causes. Treatment focuses on symptomatic management, including antipsychotics, antidepressants, and anxiolytics for psychiatric symptoms, antiepileptic drugs for seizures, and immunosuppressant-like corticosteroids for inflammation. Non-pharmacological interventions are also employed [99,100,101,102,103,104].

NPSLE is a multifaceted condition involving genetic factors, cytokines, immune cells, and environmental influences. Certain alleles of HLA genes and the TREX1 gene are associated with an increased NPSLE risk. Cytokines, crucial in immune signalling, contribute to inflammation and neurological symptoms. Elevated levels of cytokines, like IFN-γ, IL-17F, IL-21, IL-18, GM-CSF, and VEGF, are observed in patients with NPSLE, further highlighting the role of cytokines in its pathogenesis [99,100,105]. Table 1 displays the diagnosis and management of various immunological disorders, such as SCID, THI, CGD, and NPSLE.

### 7.5. Viral Infections in Children with SCID

Advancements in sequencing technologies have unveiled atypical cases of primary immunodeficiency disorders, such as JAK3 gene deficiency. A patient with chronic active Epstein–Barr virus (CAEBV) infection exhibited a poor response to ganciclovir but was diagnosed with compound heterozygous mutations in JAK3 (p.H27Q from the father; p.R222H from the mother) through next-generation and Sanger sequencing. Treatment with interferon α-2a successfully controlled the EBV and improved its symptoms. This case highlights the relevance of primary immunodeficiency considerations in CAEBV management. Interferon α-2a may serve as an effective alternative to haematopoietic stem cell transplantation in patients with JAK3 deficiency, as supported by additional cases in the literature [119].

Severe pneumonia in children presents diagnostic challenges exacerbated by non-infectious respiratory syndromes mimicking lower respiratory tract infections (LRTIs). This study utilised metagenomic next-generation sequencing (mNGS) on bronchoalveolar lavage fluid (BALF) from 126 PICU-admitted patients to identify microbial profiles. mNGS revealed diverse bacterial pathogens and indicated potential viral coinfections (Epstein–Barr virus, cytomegalovirus, and human betaherpesvirus 6B). Increased BALF bacterial diversity correlated with elevated serum inflammatory markers and lymphocyte subtype variations. These findings underscore the complexity of severe paediatric pneumonia, highlighting the role of mNGS in pathogen identification and its implications for targeted therapy and management strategies [120]. Table 2 shows the microbial pathogens associated with infections in severe combined immunodeficiency (SCID), chronic granulomatous disease (CGD), and transient hypogammaglobulinemia of infancy (THI).

In summary, while there are some overlaps in the types of microorganisms that affect patients with SCID, CGD, and THI, each immunodeficiency disorder presents unique vulnerabilities to specific pathogens due to the nature of the immune system dysfunction involved. Table 1 lists the microorganisms associated with infections in severe combined immunodeficiency (SCID), chronic granulomatous disease (CGD), and transient hypogammaglobulinemia of infancy (THI). Clinical immunology research globally encompasses a wide spectrum of immune-mediated disorders, ranging from transient hypogammaglobulinemia of infancy (THI) to severe combined immunodeficiency (SCID) and neuropsychiatric systemic lupus erythematosus (NPSLE). By unravelling the complex molecular pathways driving these disorders, clinicians and researchers collaborate worldwide to develop tailored management strategies aimed at restoring immune balance and enhancing patient outcomes. The synergy between basic science discoveries and clinical insights underscores the translational impact of immunology research in alleviating disease burden and improving the quality of life for affected individuals on a global scale [91,93,96,100].

## 8. Advancements in Cancer Research: Insights and Innovations

**Figure 3:**
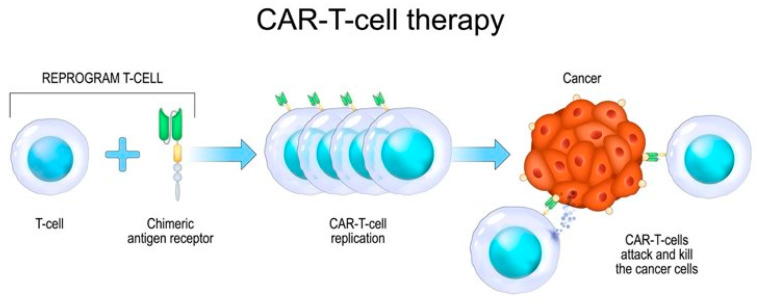
The process of CAR-T cell therapy. It has emerged as a revolutionary approach in cancer treatment, harnessing the power of the immune system to target and destroy cancer cells. The licence was purchased from shutterstock.com.

Adoptive cell transfer (ACT) has revolutionised cancer treatment, particularly through the use of tumour-infiltrating lymphocytes (TILs) against melanoma. Advances in ACT have led to the development of chimeric antigen receptor (CAR)-T cell therapy, which utilises genetically engineered T lymphocytes. CAR-T cells feature an extracellular domain derived from a monoclonal antibody’s single-chain variable fragment (scFv) for target recognition and an intracellular domain with multiple signalling motifs for T cell activation. This therapy has demonstrated remarkable success in oncology and is now being explored for other diseases. Recent trends in CAR-T cell therapy focus on its application beyond cancer, targeting autoimmune disorders and viral infections, including SARS-CoV-2, indicating its expanding potential and versatility. Traditionally, treatments for haematological cancers have included chemotherapy, radiotherapy, and stem cell transplantation. However, recent breakthroughs in tumour immunology have led to innovative therapies. One such game-changer is CAR-T cell therapy, which has shown remarkable results in relapsed/refractory B-cell acute lymphocytic leukaemia (B-ALL), non-Hodgkin lymphoma (NHL), and multiple myeloma (MM). CAR-T cell therapy involves modifying a patient’s own immune cells (T cells) to specifically target cancer cells. These modified cells are equipped with chimeric antigen receptors (CARs) that recognise specific antigens in cancer cells. Figure 3 displays the process of CAR-T cell therapy [129,130,131].

One of the key future directions of CAR-T cell therapy is the development of multi-targeted CAR-T cells. Currently, CAR-T cells are engineered to recognise a single antigen present in cancer cells. However, tumours can evade single-target therapies by downregulating the targeted antigen or developing resistance mechanisms. By designing CAR-T cells that target multiple antigens simultaneously, researchers aim to overcome tumour heterogeneity and enhance treatment efficacy. This approach not only improves tumour recognition but also reduces the likelihood of relapse, leading to more durable responses in patients [132,133].

## 9. Conclusions

The exploration of bacterial immunoglobulin-binding proteins (IBPs) and chimeric proteins marks significant advancements in immunodiagnosis, providing new approaches to combat infectious diseases and enhance patient care. Proteins such as SpA, SpG, and SpL have revolutionised serological diagnostics, offering the precise identification of bacteria, viruses, and fungi. Researchers have developed next-generation immunodiagnostic tools with enhanced specificity, stability, and accuracy, while chimeric IBPs promise early disease detection and precision medicine. Vaccine research remains crucial, particularly against HIV and tuberculosis, providing essential insights into vaccine efficacy, immunogenicity, and safety, which support global health initiatives. The rise of extended-spectrum beta-lactamases and other resistant traits highlights the need for international antimicrobial resistance surveillance to address multidrug-resistant pathogens. ESBLs confer resistance by hydrolysing the beta-lactam ring, which is essential for the bactericidal activity of these antibiotics. Variants such as SHV, TEM, and CTX-M each have unique hydrolytic capabilities. Innovations in immunological techniques have transformed disease diagnosis, blood banking practises, and transfusion medicine, improving diagnostic precision and blood safety. Clinical immunology research spans various immune-mediated disorders, offering strategies for restoring immune balance and enhancing patient outcomes.

## Figures and Tables

**Table 1:** Outline of diagnosis and management of selected immunological disorders.

Disease	Diagnosis	Management/Treatment	References
**SCID**	1. Profound deficiencies in T cells, B cells, or both at birth. 2. Due to infections, affected patients usually do not survive beyond infancy. 3. The genetic heterogeneity of SCID frequently delays diagnosis. 4. An NGS-based multigene panel for diagnosing SCID is available. 5. Other problems found in SCID are protein-losing erythroderma, alopecia, hepatosplenomegaly, lymphadenopathies, and severe diarrhoea. 6. TREC/KREC newborn screening.	1. Haematopoietic stem cell transplantation. 2. Antimicrobials. 3. Intravenous immunoglobulins. 4. Supportive therapy, such as nutritional support, aims to provide essential nutrients to maintain or improve a patient’s health. 5. Gene therapy.	[106,107,108]
**THI**	1. THI typically resolves by age four; preterm infants are especially vulnerable to THI. 2. THI, as defined by the WHO and IUIS, is a primary immunodeficiency with reduced immunoglobulin G and A levels. 3. THI diagnosis: the serum IgG levels are two standard deviations below average. 4. THI complications: recurrent infections, prolonged fever, failure to thrive, dermatitis, rhinitis, asthma, and diarrhoea. 5. The isoagglutinin levels and vaccine response are diagnostic tools for THI assessment.	1. IVIG and antibiotic prophylaxis effectively treat THI; immunotherapy reduces allergies. 2. THI can cause infections by *Staphylococcus aureus* and *Streptococcus,* treated with antibiotics like amoxicillin or amoxicillin with clavulanate, dosed by age and weight.	[109,110]
**CGD**	1. Recurrent infections by catalase-positive microorganisms like *Candida albicans* and *Staphylococcus aureus* are common in CGD. 2. Inflammatory conditions, including bowel inflammatory disease, are associated with CGD. 3. Molecular diagnosis includes next-generation sequencing (NGS), Sanger sequencing, and Genescan analysis.	1. Treatments for CGD include antibacterial prophylaxis with trimethoprim–sulfamethoxazole. Patients with sulfamethoxazole allergy have other options, such as cloxacillin and ciprofloxacin. 2. Antifungal prophylaxis with itraconazole. 3. Interferon gamma immunotherapy. 4. Haematopoietic stem cell transplantation (HCT) is the treatment of choice. 5. Gene therapy is used in a few cases.	[111,112,113,114,115]
**NPSLE**	1. NPSLE diagnosis depends on clinical signs, symptoms, lab tests, neuroimaging, and histopathology findings, tailored case by case for accuracy. 2. The presence of systemic and anti-CNS antibodies. 3. The presence of headache, psychotic manifestations, mood disorders, convulsions, and other NPSLE manifestations. 4. Testing for anti-dsDNA antibodies. 5. Complement deposition.	1. Antiepileptics, antipsychotics, anxiolytics, mood stabilisers, and antidepressants. 2. Glucocorticoids. 3. Cyclophosphamide, azathioprine, and mycophenolate mofetil. 4. Biologics: rituximab, belimumab, and anifrolumab. 5. Aspirin, heparin, and warfarin. 6. Novel oral anticoagulants: rivaroxaban, apixaban, and edoxaban.	[116,117,118]

**Table 2:** The microbial pathogens associated with infections in severe combined immunodeficiency (SCID), chronic granulomatous disease (CGD), and transient hypogammaglobulinemia of infancy (THI).

Microorganisms	SCID	CGD	THI
**Bacteria**	*S. aureus*; *Pseudomonas* spp.; *Mycobacterium bovis.* Atypical mycobacteria: *Klebsiella pneumoniae*; *Pseudomonas aeruginosa*; *Burkholderia*; *Chryseobacterium.*	*S. aureus*; *Nocardia* spp.; *Burkholderia* spp.; *Serratia* spp.; *Chromobacter* spp.; *Salmonella* spp.	*Streptococcus pneumoniae*; *Haemophilus*; *influenzae* type b; *Pseudomonas aeruginosa*; *S. aureus*; *Clostridium difficile.*
**Viruses**	Cytomegalovirus; Adenovirus; Enterovirus; Herpes simplex virus; Respiratory syncytial virus; Epstein–Barr virus; Rotavirus; Parainfluenza virus.	It is not a primary concern.	Respiratory syncytial virus; Enteroviruses; Rotavirus.
**Fungi**	*Pneumocystis jirovecii*; *Histoplasma capsulatum*; *Cryptococcus neoformans*; *Candida albicans*; *Aspergillus* spp. *Acremonium*; *Pichia.*	*Aspergillus* spp.; *Candida* spp.; *Fusarium dimerum*; *Penicillium*; *Paecilomyces variotii*; *Scedosporium.*	*Candida* spp.
**Parasites**	*Giardia duodenalis*; *Giardia intestinalis*; *Cryptosporidium* spp.; *Schistosoma* species; *Blastocystis hominis*; *Fasciola* spp.; *Trichostrongylus* spp. *Cryptosporidium* spp.	It is not a primary concern.	*Giardia lamblia.*
**References**	[121,122,123]	[124,125,126]	[127,128]

## Data Availability

The dataset supporting the findings of this study is included within the manuscript and its referenced sources, ensuring comprehensive access to the relevant data for further examination and analysis.
